# Restless Legs Syndrome in Patients with Psoriatic Arthritis: Association with Inflammatory and Clinical Parameters and Other Comorbidities—A Cross-Sectional Observational Study

**DOI:** 10.3390/biomedicines13123028

**Published:** 2025-12-10

**Authors:** Esther Toledano, Luis López-Mesonero, Javier Martín-Vallejo, Carolina Cristina Chacón, Roberto Díaz-Peña, Pilar Sánchez-Conde, Daniel Martín, Cristina Hidalgo, Sergio Cimadevila, Carlos Montilla

**Affiliations:** 1 Department of Rheumatology, San Carlos Clinical Hospital, 28040 Madrid, Spain; esthertoledano@hotmail.com; 2 Department of Neurology, Clinical University Hospital of Salamanca, 37007 Salamanca, Spain; id00494988@usal.es; 3 Statistics Department, Universidad de Salamanca, 37007 Salamanca, Spain; jmv@usal.es; 4 Department of Rheumatology, Clinical University Hospital of Salamanca, 37007 Salamanca, Spainchidalgoc15@gmail.com (C.H.); sergiocima66@gmail.com (S.C.); 5 Fundación Pública Galega de Medicina Xenómica, SERGAS, Grupo de Medicina Xenómica-USC, Health Research Institute of Santiago de Compostela (IDIS), 15700 Santiago de Compostela, Spain; roberto.diaz.pena@sergas.es; 6 Faculty of Health Sciences, Universidad Autónoma de Chile, Cinco Poniente 1670, Talca 3460000, Chile; 7 Department of Anesthesiology, Clinical University Hospital of Salamanca, 37007 Salamanca, Spain; pconde@usal.es; 8 Gerencia de Atención Primaria, Valladolid Oeste, 47012 Valladolid, Spain; dmartinh96@gmail.com; 9 Department of Medicine. Faculty of Medicine, University School of Medicine, 37007 Salamanca, Spain

**Keywords:** psoriatic arthritis, restless legs syndrome, sleep quality, anxiety, depression, fatigue

## Abstract

**Introduction/Objectives:** Restless legs syndrome (RLS), a chronic neurological disorder related to brain iron metabolism, has been linked to immune-mediated inflammatory conditions such as psoriatic arthritis (PsA). However, the role that inflammation plays in this association and the impact of RLS on PsA outcomes remain unclear. This study aims to investigate the association between RLS and inflammatory/clinical parameters in PsA patients. **Materials and Methods:** In this cross-sectional study, 230 PsA patients completed the International Restless Legs Syndrome Study Group (IRLSSG) screening questionnaire, with diagnoses confirmed by a neurologist. Data collected included clinical features, disease activity, and comorbidities (obesity, anxiety, depression, insomnia, and fibromyalgia). **Results:** In total, forty-six patients met the IRLSSG criteria (20%). Those with RLS more frequently had polyarthritis (27% vs. 6%; *p* < 0.001), more swollen joints (2.0 vs. 1.4; *p* = 0.04), greater psoriatic involvement (5.7 vs. 3.6; *p* < 0.001), greater fatigue (39.0 vs. 30.5; *p* < 0.001), and greater disease activity (14.5 vs. 10.5; *p* < 0.001). They also exhibited increased disease impact (4.7 vs. 2.9; *p* < 0.001), poorer functioning (0.7 vs. 0.5; *p* = 0.01), and higher levels of anxiety (8.0 vs. 5.5; *p* < 0.001), depression (6.5 vs. 3.9; p < 0.001), and sleep disturbance (13.9 vs. 8.7; *p* < 0.001). Skin lesions and polyarthritis explained nearly 40% of RLS cases (Odds Ratio (OR) 1.4; 95% Confidence Interval (CI) 1.03–2.0; *p* = 0.03 and OR 1.03; 95% CI 1.00–1.9; *p* = 0.04). **Conclusions:** Psoriatic activity and inflammation may contribute to RLS in PsA. The coexistence of RLS was associated with greater disease activity, greater disease impact, and more emotional and sleep-related comorbidities.

## 1. Introduction

Psoriatic arthritis (PsA) is a chronic inflammatory disease that can affect the joints, entheses, the spine, and the skin. Psoriatic arthritis is a chronic, immune-mediated inflammatory arthritis associated with psoriasis. Its incidence varies geographically, influenced by genetic and environmental factors. Genetic predisposition plays a significant role, with familial aggregation and specific HLA alleles contributing to susceptibility. The pathogenesis is complex and multifactorial, involving an interplay between genetic susceptibility and environmental triggers such as infections, microbiota changes, obesity, and biomechanical stress, which activate innate and adaptive immune responses [1].

It is associated with a wide range of comorbidities, including sleep disorders [2]. These disorders are a heterogeneous group of conditions, of which sleep apnoea, insomnia, and restless legs syndrome (RLS) are the most common.

RLS is a chronic movement disorder characterised by an urge to move one’s legs, generally accompanied by uncomfortable feelings, that may interfere with sleep. Although the physiopathology of RLS is not fully understood, the most widely accepted hypotheses include (a) genetic variants; (b) abnormal iron metabolism in the central nervous system; and (c) dopaminergic dysfunction [3]. The decision to investigate the association between RLS and PsA is supported by several converging lines of evidence. First, emerging research indicates that RLS is significantly more prevalent in patients with immune-mediated inflammatory diseases, including rheumatoid arthritis (up to 30%), systemic lupus erythematosus, and axial spondyloarthritis, compared to the general population (7–10%). Second, both conditions share common pathophysiological mechanisms involving systemic inflammation and immune dysregulation. Multiple studies have demonstrated elevated inflammatory markers, particularly C-reactive protein (CRP), tumour necrosis factor-alpha (TNF-α), and interleukin-17 (IL-17), in patients with RLS. These same pro-inflammatory cytokines play central roles in PsA pathogenesis, suggesting overlapping inflammatory pathways that could mechanistically link these conditions [4,5,6,7,8]. Nonetheless, there is currently limited information on the influence of the inflammatory process on the development of RLS or the impact of RLS on clinical manifestations in PsA. Because of this, we decided to perform a study that assesses the relationship of RLS with inflammatory and clinical factors in a cohort of patients with PsA.

## 2. Materials and Methods

We performed a cross-sectional observational study that included adults (over 18 years of age) diagnosed with PsA according to the CASPAR criteria [9]. Participants who agreed to participate in this study and gave their written informed consent were consecutively recruited from the Rheumatology Department at Salamanca University Hospital between 1 September 2023 and 30 April 2024.

Exclusion criterion: Patients taking antidepressant or anxiolytic drugs, given their potential influence on the occurrence of RLS [10,11].

**Figure 1:**
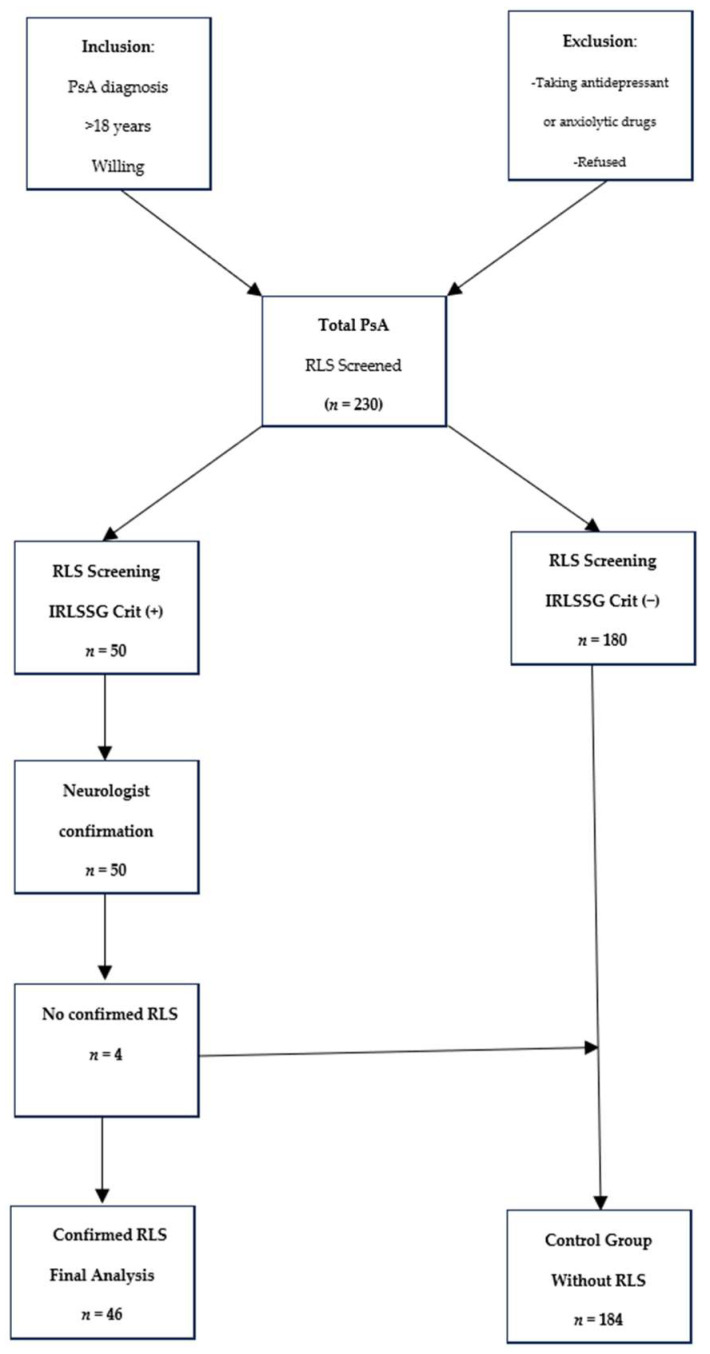
Study Population Flow Diagram and Selection Process. +: Positive, −: Negetive.

All participants completed a screening questionnaire based on the International Restless Legs Syndrome Study Group (IRLSSG) criteria [12]. This questionnaire covered four features of RLS: (1) the urge to move the legs, usually but not always accompanied by—or felt to be caused by—uncomfortable and unpleasant sensations in the legs; (2) the urge to move the legs and any accompanying unpleasant sensations that begin or worsen during periods of rest or inactivity, such as lying down or sitting; (3) the urge to move the legs and any accompanying unpleasant sensations that are partially or totally relieved by movement, such as walking or stretching, at least as long as the activity continues; and (4) the urge to move the legs and any accompanying unpleasant sensations during rest or inactivity that only occur or are worse in the evening or at night than during the day. Patients who reported showing all four of these features were assessed by an expert neurologist (LLM) who confirmed or ruled out the diagnosis of RLS. The process is summarised in the flowchart (Figure 1).

### 2.1. Data Were Recorded on the Following Variables

#### 2.1.1. Standard Baseline Demographic Characteristics and Routine Blood Test Results

Age, sex, time since disease onset (years), smoking status (smoker/former smoker/non-smoker; a person who had previously been a smoker but had not smoked for at least 12 months was classified as a former smoker), and the number of cigarettes smoked measured in pack-years [13]; CRP level, additional blood parameters such as iron status (iron, ferritin, transferrin saturation, total iron-binding capacity), and kidney function (creatinine level) due to their potential association with RLS.

#### 2.1.2. Variables Related to the Treatment Received

Treatment with conventional synthetic disease-modifying antirheumatic drugs (csDMARDs), targeted synthetic disease-modifying antirheumatic drugs (tsDMARDs), and biologic disease-modifying antirheumatic drugs (bDMARDs), and also any changes in treatment due to poor response to tsDMARDs or bDMARDs.

#### 2.1.3. Variables Related to Psoriatic Disease

Clinical form of the disease at the time of the study peripheral, mixed, or axial classifying patients with inflammatory lower back pain and radiographic damage (sacroiliitis of at least grade 2 as per New York criteria and/or the presence of syndesmophytes) as having axial involvement [14,15]; dactylitis (current or past); and the number of entheses involved as assessed using the modified Maastricht Ankylosing Spondylitis Enthesitis Score (mMASES) [16]. The original MASES [17] took into account 13 entheses (the bilateral first and seventh costochondral joints, the anterior and posterior superior iliac spine, the iliac crests, the proximal insertion of the Achilles tendons, and the fifth lumbar spinous process). That score was modified for PsA to include the plantar fascia, with scores ranging from 0 to 15 [17]. Further, the extent of psoriasis was assessed using the Psoriasis Area Severity Index (PASI) [18] and the third item of the PsA Impact of Disease (PsAID) questionnaire (skin problems, including itchiness), and *fatigue* was assessed using the Functional Assessment of Chronic Illness Therapy (FACIT) fatigue scale [19]. Permission to use the latter instrument was obtained from FACIT.org (Access date: 22 October 2023).

Disease activity, functioning, and disease impact: In the case of peripheral involvement, disease activity was measured using the Disease Activity in Psoriatic Arthritis (DAPSA) score [20], a composite measure designed to assess disease activity in PsA. The DAPSA score is the sum of the CRP (mg/dL), tender joint count (0–68), swollen joint count (0–66), the patient’s global assessment of disease activity score (between 0 and 10 on a numerical rating scale [NRS]), and the pain NRS score (0–10). In addition, we recorded whether patients had polyarthritis (involvement of five or more joints). In cases of axial involvement, we used the Ankylosing Spondylitis Disease Activity Score with C-reactive protein (ASDAS-CRP) [21]. We measured functional ability based on the Health Assessment Questionnaire-Disability Index (HAQ-DI) for peripheral involvement and the Bath Ankylosing Spondylitis Functional Index (BASFI) for axial involvement [22,23]. Disease impact was assessed using the PsAID-12 [24].

#### 2.1.4. Further, Comorbidities Were Evaluated Using Validated Questionnaires

Emotional distress was assessed using the Hospital Anxiety and Depression Scale (HADS), a 14-item scale applied to identify anxiety and depression in patients with physical illnesses. Scores range from 0 to 21 for each subscale (HADS-D for depression and HADS-A for anxiety) and are classified as normal (0–7); borderline abnormal, indicating a possible clinical disorder (8–10); and abnormal, indicating a probable clinical disorder (11–21) [25].

Sleep quality was measured with the Insomnia Severity Index (ISI). The ISI is a self-administered questionnaire that includes 7 items that address the nature, severity, and impact of insomnia. Patient responses are reported on a 5-point Likert-type scale (0 to 4) for the previous month. The overall score ranges between 0 and 28 and is classified as no clinically significant insomnia (0–7), subthreshold insomnia (8–14), clinical insomnia (moderate severity) (15–21), and clinical insomnia (severe) (22–28) [26].

Obesity was measured using body mass index (BMI) [27].

Fibromyalgia: This condition was assessed using the diagnostic criteria of the Analgesic, Anaesthetic, and Addiction Clinical Trial Translations, Innovations, Opportunities, and Networks—American Pain Society Pain Taxonomy (AAPT) [28].

### 2.2. Statistical Analysis

Quantitative variables are reported as the mean and standard deviation (SD) if the data are normally distributed; otherwise, they are reported as the median and interquartile range (IQR), and the categorical variables are reported as numbers and percentages (N/%). The groups were compared using the *t*-test for normally distributed quantitative variables and the Mann–Whitney test for ordinal or non-normally distributed quantitative variables. Comparisons between more than two groups were performed using one-factor analysis of variance (normally distributed quantitative variables) and the Kruskal–Wallis test (ordinal or non-normally distributed quantitative variables). The threshold for statistical significance was set at *p* < 0.05.

Binary logistic regression explanatory model was employed to identify independent predictors of RLS occurrence in PsA patients. This model is appropriate for binary dependent variables (presence/absence of RLS), where the outcome is coded as 1 (RLS confirmed) or 0 (no RLS). These included both variables found to be significant in our univariate analysis and those associated with RLS in previous studies, such as fatigue [6], psoriasis severity [29], CRP [30,31], ferritin levels [32], smoking status [33,34], age [35], obesity [36], sex [12], and fibromyalgia [37].

Two approaches were used to analyse the reliability and stability of the logistic regression results in relation to the sample size of the study. First, the approach proposed by Peluzzi et al. [38] was applied, whereby the sample size for a logistic regression is calculated based on the events per variable, the number of predictor variables, and the expected proportion. The adjusted logistic model has 12 variables, a minimum of 10 events per variable, and an expected proportion of 0.5, so the required sample size would be 240. The power has also been calculated for a logistic regression model with a dichotomous variable, a significance level of 5%, an odds ratio of 2, and a proportion of 50%. Under these conditions, the power of the analysis would be 80%. The ‘powermediation’ package, version 0.3.4, in R, version 4.5.2, was used to calculate the statistical power of the models. Statistical analyses were performed using IBM SPSS Statistics for Windows, Version 23.0 (IBM Corp., Armonk, NY, USA).

### 2.3. Ethical Statement

#### 2.3.1. Study Approval and Ethical Compliance

This study was conducted in accordance with the principles established in the Declaration of Helsinki and adhered to all applicable ethical guidelines for human research. The study protocol was prospectively reviewed and approved by the Institutional Review Board (Ethics Committee) of Salamanca University Hospital (Comité de Ética en Investigación Clínica—CEIC), with project number PI 2023 03 1560 (approved on 24 April 2023).

#### 2.3.2. Informed Consent Procedures

All eligible participants were provided with detailed written information about the study objectives, procedures, data collection methods, and potential risks or benefits of participation. Prior to enrollment, written informed consent was obtained from each participant after ensuring they fully understood the study protocol and had the opportunity to ask questions. All participants had the right to withdraw from the study at any time without providing justification or incurring any consequences on their clinical care.

#### 2.3.3. Data Protection and Confidentiality

Patient data were collected and managed in accordance with current data protection regulations. All collected information was assigned a unique identification code, and personal identifiers were removed from the database to ensure participant confidentiality and anonymity throughout the study. Data were stored securely in a password-protected database accessible only to authorised research team members.

## 3. Results

### 3.1. Baseline Variables

Out of the 230 patients recruited, 50 (21.7%) met the IRLSSG criteria for RLS [12]. After being assessed by a neurologist (LM), four patients were excluded from this study (three with a diagnosis of nocturnal cramps and one with polyneuropathy).

Among the 46 patients with confirmed RLS (20% of the sample), the majority were smokers, and this group had more discomfort associated with psoriasis and reported greater levels of fatigue. Table 1 summarises our analysis of these and other descriptive statistics.

### 3.2. Disease Activity, Functioning, and Disease Impact

Patients with RLS were found to have greater disease activity clinically in terms of both swollen and tender joint counts. Moreover, they also had poorer functioning. The results are shown in Table 2.

### 3.3. Comorbidities

Patients with RLS had higher levels of anxiety and depression and poorer sleep quality. The results are shown in Table 3.

In binary logistic regression explanatory model, Nagelkerke R^2^: 0.38, skin problems/itchiness (OR 1.4; 95% CI 1.03–2.0; *p* = 0.03) emerged as the only robust independent predictor of RLS. Although polyarthritis (presence/absence) (OR 1.03; 95% CI 1.00–1.9; *p* = 0.04) reached nominal statistical significance, the confidence interval boundary at 1.0, minimal effect size (3% odds increase), and small number of polyarthritis cases (*n* = 12) suggest this finding is marginally significant and warrants cautious interpretation. None of the other variables reached significance: FACIT-F (*p* = 0.7), PASI (*p* = 0.8), CRP (*p* = 0.1), ferritin (*p* = 0.2), HADS-D (*p* = 0.1), smoking exposure in pack-years (*p* = 0.2), age (*p* = 0.5), BMI (*p* = 0.1), sex (*p* = 0.06), or fibromyalgia (*p* = 0.3).

## 4. Discussion

RLS that occurred alongside PsA was associated with more symptomatic psoriasis (related to itching). It is possible that the polyarticular inflammatory process may increase the presence of this comorbidity.

As only a few studies within the scientific literature have examined the relationship between PsA and RLS, it is difficult to compare our results with those of previous research. Indirect evidence from a small cohort of patients with PsA was provided by Sandikci et al., who observed a higher rate of DMARD use in patients with RLS than those without RLS, suggesting that individuals with this syndrome had greater skin and joint disease activity [8].

In our study, skin problems (including itchiness) were related to the presence of RLS. To our knowledge, this association has not previously been reported, although itchiness has previously been associated with poorer sleep quality in patients with cutaneous psoriasis. In Callis Duffin et al.’s study, itchiness was a predictor of poor sleep quality (OR 1.24, 95% CI 1.11-1.39; *p* < 0.0001) [39]. It is possible to hypothesise that abnormalities in dopamine pathways, related to the aetiopathogenesis of RLS, may be responsible for sensory abnormalities that increase the discomfort associated with psoriasis. Previous studies have already observed a relationship between psoriasis severity and RLS. In particular, Solak et al. found an association between psoriasis severity, as measured by the PASI, and the development of RLS. The authors attributed this association to the greater levels of inflammation seen in the most severe forms of psoriasis. We did not find a relationship between PASI and RLS: this difference may be due to our patients having milder cutaneous involvement (1.8 vs. 5.4 in patients studied by Solak et al.) [29].

With the limitations related to the OR value, we found a possible relationship between the joint inflammatory process and the presence of SPI. In patients with psoriasis, Solak et al. concluded that RLS was associated with higher levels of CRP (5.0 vs. 3.0 mg/dL; *p* = 0.03) [29]. Their results are in line with those of Nowowiejska et al., who, in a cohort of 70 patients with psoriasis, also found that patients with RLS had higher levels of CRP (*p* = 0.01) [30]. In another context, in a recently published paper, Dowsett et al. compared plasma from donors who were and were not classified as RLS cases and found that those with RLS had higher CRP levels than the controls (0.74 mg/L, IQR: 0.17–1.90 vs. 0.52 mg/L, IQR: 0.14–1.39; *p* = 0.032) [31]. Further, when CRP plasma concentrations were grouped into high (>3 mg/L) and low (<3 mg/L) levels, a higher percentage of RLS cases compared with the controls had high CRP (*p* = 0.049) [31]. Nonetheless, similar studies have yielded different results [30,40], though these inconsistencies may be due to the use of different criteria to diagnose RLS. Despite this indirect evidence, the role of inflammation in the physiopathology of RLS is not yet fully understood. This role may be able to be explained given the link between inflammation and hypoxia. A proteomics study found that eight inflammation-related proteins were differentially expressed in patients with RLS, and network analysis revealed indirect links with the proteins involved in hypoxia pathways [41].

Regarding other variables potentially associated with RLS, our univariate analysis indicated that patients with RLS had higher levels of fatigue, anxiety, and depression, as well as poorer sleep quality. In the multivariate analysis, Sandikci et al. found that RLS severity was associated with fatigue and depression [8]. An association is very commonly found between fatigue and RLS. Nonetheless, as the physiopathology of these conditions is not fully understood, the reasons for such an association remain unknown, although it may be related to abnormalities in dopamine pathways [42,43].

In a recent systematic review, depression was found to be associated with RLS (OR = 1.71, 95% CI = 1.26–2.32) [35]. Although the precise mechanisms underlying this association have yet to be established, there is some indirect evidence that may help explain it. On the one hand, RLS results in poor sleep quality, which, in turn, may lead to the development of anxiety and/or depression [44]; on the other hand, lower levels of monoamine neurotransmitters, such as dopamine, may be associated with increases in anxiety and depression [45].

In our study, PsA patients with RLS had poorer functioning and greater disease impact. We have not found data with which to compare these findings due to the scarcity of studies assessing RLS in patients with PsA.

In the analysis of baseline characteristics, patients with RLS were not significantly more likely to be smokers (current or former) than never smokers, nor was a statistically significant difference reached when we considered smoking exposure in pack-years. However, a recent meta-analysis found an association between smoking and RLS (OR = 1.46; 95% CI = 1.29–1.64). The mechanism underlying this association remains unknown but may be related to complications in blood circulation in people with smoking habits [34]. We found no associations with other variables studied that have been found to be associated with RLS in previous research (sex, age, BMI, and fibromyalgia) [35,36].

The most important limitation of this study is likely its cross-sectional nature. This limitation is common in studies of RLS and is attributable to the risk factors for this syndrome not yet being well defined. Indeed, we are not able to establish causal relationships. For future studies, we are currently following up with this group of patients to detect cases of incident restless legs syndrome. Nonetheless, based on our study, motivated by previous data suggesting that inflammation is a potential trigger for RLS, we can state that our results support this hypothesis; that is, the marked inflammation seen in polyarthritis may play a role in the development of RLS. On the other hand, we could speculate that, in patients with RLS, greater levels of skin problems, related to psoriasis, may be due to an abnormal sensory response caused by an alteration in dopamine pathways [43]. Another limitation of this study was that we did not examine the severity of RLS symptoms, meaning that we could not explore potential correlations with inflammation severity, nor did we include the SPI evolution time in the data. Finally, the inclusion of a control group without PsA would allow for comparison of RLS prevalence between patients and healthy individuals, strengthening the study’s conclusions.

The main strength of this study was that the diagnosis of RLS was made based on IRLSSG criteria and subsequently confirmed by a neurologist. The symptoms associated with RLS are common to various neurological diseases; hence, the need for a definitive diagnosis by a specialist [46]. Another strength is the inclusion of several variables (smoking habits, depression, and fatigue) that had not previously been associated with RLS in patients with PsA.

## 5. Conclusions

This cross-sectional study provides evidence that restless legs syndrome (RLS) is a relevant comorbidity in patients with psoriatic arthritis (PsA), with a prevalence of 20% in our cohort. The main findings can be summarised as follows:

Psoriatic skin symptoms, particularly itchiness, were independently associated with RLS, suggesting that sensory abnormalities—possibly related to dopaminergic pathway dysfunction—may amplify discomfort in patients with cutaneous psoriasis.

Polyarticular joint inflammation showed a marginal association with RLS, although this finding should be interpreted cautiously due to the borderline confidence interval and small effect size. Nevertheless, these results support the hypothesis that systemic inflammation may contribute to the development of RLS in PsA.

## Figures and Tables

**Table 1:** General characteristics of the disease and treatment received overall and stratified by whether patients met the RLS criteria.

Variable	All (*n* = 230)	RLS (*n* = 46)	No RLS (*n* = 184)	*p*
Age, years *	54.6 ± 11.3	51.3 ± 11.9	53.4 ± 11.2	0.2
Sex (woman/man)	104/126	24/22	80/104	0.2
Time since onset of PsA symptoms (years) **	8 (9)	8 (9)	10 (10)	0.9
Smoking status, N (%)				
Smoker (current or former)	166 (72)	33 (72)	133 (72)	0.9
Never smoker	64 (28)	3 (28)	51 (28)	
Smoking, pack-years *	12.9 ± 20	18.0 ± 18.6	14.5 ± 9.2	0.3
Iron (mcg/dL) **	87.0 (43)	88.0 (42)	87.0 (43)	0.8
Ferritin (ng/mL) **	122.0 (163)	93 (131)	133 (168)	0.2
Transferrin saturation (%) *	27.2 ± 13.9	27.5 ± 14.4	26.9 ± 13.7	0.6
Total iron-binding capacity (mcg/dL) **	325 (66)	316 (47)	328 (65)	0.07
Creatinine(mg/dL) **	0.8 (0.2)	0.8 (0.2)	0.8 (0.2)	0.6
Conventional synthetic DMARDs, N (%)	170 (74)	36 (78)	134 (73)	0.7
Methotrexate	129 (56)	29 (63)	100 (54)	
Sulfasalazine	32 (14)	5 (11)	27 (15)	
Leflunomide	9 (4)	2 (4)	7 (4)	
tsDMARDs or bDMARDs, N (%)	64 (29)	16 (25)	48 (28)	0.8
TNF inhibitor	47 (17)	11 (14)	36 (19)	
Secukinumab	10 (7)	2 (8)	8 (6)	
Ustekinumab	3 (1)	2 (1)	1 (2)	
Tofacitinib	3 (2)	2 (3)	1 (1)	
Apremilast	2 (1)	0	2 (1)	
Failure of tsDMARDs or bDMARDs, N (%)	28 (12)	16 (17)	12 (11)	0.2
Clinical presentation, N (%)				
Peripheral	194 (85)	37 (80)	157 (85)	0.6
Mixed	30 (12)	7 (15)	23 (13)	
Axial	6 (3)	2 (5)	4 (2)	
Dactylitis (yes/no) (%)	36/194 (15)	3/43 (6)	33/151 (18)	0.07
mMASES *	1.4 ± 2.4	1.8 ± 2.5	1.3 ± 2.3	0.2
PASI *	1.8 ± 2.6	1.7 ± 2.2	1.8 ± 2.7	0.9
PsAID (Item 3) *	4.0 ± 3.0	5.7 ± 2.1	3.6 ± 2.9	0.001
FACIT-F **	38.0 (16)	30.5 (21)	39.0 (13)	0.001

Abbreviations: DMARD: disease-modifying antirheumatic drug; tsDMARD: targeted synthetic disease-modifying antirheumatic drug; bDMARD: biologic disease-modifying antirheumatic drug; mMASES: modified Maastricht Ankylosing Spondylitis Enthesitis Score; PASI: Psoriasis Area Severity Index; PsAID: Psoriatic Arthritis Impact of Disease; FACIT-F: Functional Assessment of Chronic Illness—Fatigue. * Mean/SD; ** Median/IQR. All laboratory parameters were measured using standard clinical chemistry methods at Salamanca University Hospital’s clinical laboratory.

**Table 2:** Disease activity, functioning, and disease impact.

Variable	All (*n* = 230)	RLS (*n* = 46)	No RLS (*n* = 184)	*p*
DAPSA *	14.9 ± 7.4	14.5 ± 7.2	10.5 ± 6.3	0.001
CRP (mg/dL)	0.3 ± 0.5	0.3 ± 0.5	0.2 ± 0.3	0.10
TJC	1.5 ± 2.0	2.6 ± 3.0	1.3 ± 1.6	0.007
SJC	1.5 ± 1.4	2.0 ± 1.5	1.4 ± 1.3	0.04
Activity VAS	4.2 ± 2.7	4.5 ± 2.5	4.0 ± 2.7	0.2
Pain VAS	4.0 ± 2.7	4.9 ± 2.6	3.8 ± 2.7	0.01
Polyarthritis (yes/no) (%)	23/201 (10)	12/32 (27)	11/169 (6)	0.001
ASDAS-CRP **	1.7 ± 0.8	2.0 ± 0.8	2.0 ± 0.9	0.7
HAQ-DI *	0.6 ± 0.6	0.7 ± 0. 6	0.5 ± 0.5	0.01
BASFI **	3.5 ± 2.8	4.7 ± 2.9	3.3 ± 2.7	0.2
PsAID-12	3.3 ± 2.1	4.7 ± 2.0	2.9 ± 2.0	0.001

Abbreviations: DAPSA: Disease Activity Index for Psoriatic Arthritis; C-reactive protein; TJC: tender joint count; SJC: swollen joint count; VAS: visual analogue scale; ASDAS-CRP: Ankylosing Spondylitis Disease Activity Score with C-reactive protein; HAQ-DI: Health Assessment Questionnaire-Disability Index; BASFI: Bath Ankylosing Spondylitis Functional Index; PsAID: Psoriatic Arthritis Impact of Disease. * In peripheral and mixed forms (*n* = 224). ** In axial and mixed forms (*n* = 45).

**Table 3:** Comorbidities (emotional state, sleep quality, obesity, and fibromyalgia).

Variable	All (*n* = 230)	RLS (*n* = 46)	No RLS (*n* = 184)	*p*
HADS-A	6.3 ± 4.0	8.0 ± 4.7	5.5 ± 3.5	0.001
HADS-D	4.4 ± 3.7	6.5 ± 4.6	3.9 ± 3.2	0.001
ISI	9.7 ± 6.0	13.9 ± 6.9	8.7 ± 6.0	0.001
BMI (kg/m^2^)	27.2 ± 5.0	27.4 ± 5.3	26.4 ± 3.4	0.3
Fibromyalgia (yes/no) (%)	14/216 (6)	5/41 (11)	9/175 (5)	0.1

Abbreviations: HADS-A and HADS-D: Hospital Anxiety and Depression Scale Anxiety and Depression subscales, respectively; ISI: Insomnia Severity Index; BMI: body mass index.

## Data Availability

The data presented in this study are available upon request from the corresponding author.
